# Mapping the Extended Pain Pathway: Human Genetic and Multi-Omic Strategies for Next-Generation Analgesics

**DOI:** 10.3390/ijms27073035

**Published:** 2026-03-26

**Authors:** Ari-Pekka Koivisto

**Affiliations:** Orion Pharma, Orion Corporation, FI-20101 Turku, Finland; ari-pekka.koivisto@orionpharma.com

**Keywords:** human genetics, human pain, NaV1.7, NaV1.8, TRPA1, target validation, pain therapeutics, Suzatrigine, VX993, LY3526318

## Abstract

The 2025 approval of the selective NaV1.8 blocker suzetrigine for acute pain marked a pivotal advance in analgesic drug development. Yet the subsequent failure of Vertex’s next-generation NaV1.8 inhibitor VX993 to demonstrate clinical analgesia underscores enduring challenges in translating mechanistic promise into patient benefit. This review examines why promising targets and compounds, spanning NaV and TRP channels, often falter and outlines a path toward more reliable target selection and validation. I first summarize the pain pathway, from nociceptor transduction through spinal processing to cortical perception, emphasizing how inflammation and peripheral sensitization reshape excitability. Historically serendipitous, pain drug discovery now prioritizes molecular precision. Most approved chronic pain therapies act in the CNS and are limited by modest efficacy and adverse effects. Nociceptor-enriched targets (NaV1.7/1.8/1.9; TRP channels) remain attractive, yet redundancy among NaV subtypes and the necessity of blocking targets at the correct anatomical sites complicate translation. Human genetics and multi-omics provide a powerful, unbiased engine for target discovery. Rare high-impact variants offer strong causal hypotheses, while common polygenic contributions illuminate broader susceptibility. Large biobanks increasingly reveal a mismatch between legacy pain targets and genetically supported candidates across neuronal and non-neuronal cells. Human DRG transcriptomics highlight NaV channel redundancy. Human in vitro electrophysiology and PK/PD analyses show suzetrigine achieves ~90–95% NaV1.8 engagement, yet neurons can still fire unless additional channels are blocked. Species differences and drug distribution (including BBB/PNS penetration and P-gp efflux) critically influence efficacy; centrally accessible blockade (e.g., for NaV1.7 or TRPA1) may be necessary to achieve robust analgesia, challenging peripherally restricted strategies. Osteoarthritis illustrates how obesity-driven metabolic inflammation, synovial immune activation, subchondral bone remodeling, and specific nociceptor subtypes converge to drive mechanical pain. Multi-omic integration across diseased human tissues can pinpoint causal processes and cell types, enabling more selective and safer target choices. I propose a practical framework for target validation that integrates: (i) rigorous human genetic support; (ii) cell-type and site-of-action mapping; (iii) human-relevant electrophysiology and PK/PD with verified target engagement; (iv) species-appropriate models; (v) consideration of modality (small molecule, biologic, RNA, targeted protein degradation). Advancing genetically and anatomically aligned targets, tested at the right sites and exposures, offers the best path to genuinely effective, better-tolerated pain therapeutics.

## 1. Introduction

The approval of the selective NaV1.8 channel blocker Suzetrigine for acute pain treatment in 2025 marked a major milestone in the long quest to develop novel pain-relieving drugs [1,2]. This success raised hopes that more new pain drugs, targets, and novel modalities would soon follow. However, looking back, many once-promising targets never reached clinical application.

Adding to the complexity, Vertex, the company behind Suzetrigine, recently announced that its next-generation NaV1.8 blocker, VX993, failed to demonstrate clinical analgesia in acute pain [3]. This raises two critical questions:

- Why did VX993 fail?
- Why is developing new pain drugs so challenging?

## 2. The Pain Pathway and Modern Drug Targets for Pain Relief

The pain pathway relies on tightly coordinated interactions among nociceptors, peripheral sensory neurons that specialize in detecting noxious stimuli, the spinal cord, and the brain. Pain perception begins at the free nerve endings of these sensory neurons, where specialized ion channels, such as ASIC, HCN, and TRP channels, and a variety of receptors detect harmful mechanical, chemical, or thermal cues. These molecular sensors convert damaging stimuli into electrical signals by depolarizing the neuronal membrane potential.

The coordinated activity of several depolarizing and repolarizing ion channels can generate rhythmic oscillations in the membrane potential. Once the depolarization reaches a critical threshold, the NaV1.7 sodium channel triggers an action potential.

The intensity of pain is encoded by the frequency of these action potentials; the faster the firing, the stronger the perceived pain. The NaV1.8 channel plays a key role in sustaining high-frequency action potential firing, enabling the peripheral nervous system to transmit signals that correspond to more intense pain.

During inflammation or tissue injury, immune cells migrate to the affected site and release mediators that sensitize nociceptors. As a result, previously harmless stimuli may become painful, and painful stimuli may intensify, a process known as peripheral sensitization.

From the periphery, pain signals travel as action potentials through voltage-gated sodium channels into the spinal cord. There, voltage-gated calcium channels regulate the release of glutamate and neuropeptides, relaying the signal to second-order neurons. Interneurons in the spinal dorsal horn further amplify or dampen this signal. Finally, lamina I neurons project the information to the brain, where pain is experienced as an unpleasant sensation [4].

## 3. Evolution of Pain-Relief Drug Discovery

Historically, pain-relieving compounds were discovered largely by chance, through serendipitous findings, the use of plant extracts, animal-derived substances, and empirical trial-and-error testing in humans. Modern drug discovery, however, has shifted toward a more precise and rational approach. Today, well-defined molecular targets guide the development process, with selective small-molecule modulators remaining a central and highly effective strategy for advancing new analgesics.

Today, most pain-drug development is conducted by private pharmaceutical companies and academic research groups around the world. Despite differences in mission and structure, both face the same fundamental challenge: how to use limited resources wisely while identifying the most promising drug targets.

The aim of this review is to offer insight not only into the selection of a drug target, but also into the demanding process of validating that target as truly meaningful. In many cases, compounds or projects that initially failed can provide crucial information that reshapes our understanding of target validity and may even revive interest in molecules or mechanisms once considered unpromising.

My hypothesis is that drug targets chosen on the basis of human genetic evidence, combined with a precise understanding of the site of drug action, may be far more important than previously recognized. In this review, I illustrate this idea with several recent examples.

Many approved chronic pain medications, such as opioids, gabapentinoids, SNRIs, and tricyclic antidepressants, work primarily within the central nervous system (CNS). While these drugs can offer relief, their benefits often come at the cost of significant CNS-related side effects, including sedation, dizziness, hallucinations, and addiction. Because of these risks, the ideal analgesic targets would be highly selective for nociceptors, allowing effective pain relief without disrupting broader brain function.

Compounding the challenge, the analgesic efficacy of existing chronic pain treatments remains modest. This is often illustrated using the Number Needed to Treat (NNT), a standard metric in meta-analyses of pain clinical trials. NNT represents how many patients must receive a therapy for just one subject to achieve a clinically meaningful reduction in pain. Lower NNT values indicate stronger efficacy, but for many current pain medications, NNTs are relatively high, underscoring the unmet need for more effective analgesics.

Sodium channels NaV1.7, NaV1.8, and NaV1.9, along with several TRP channels, are expressed predominantly in nociceptors, making them exceptionally attractive targets for next-generation analgesics. Their central role in controlling neuronal excitability provides a strong mechanistic rationale for developing compounds that block channel activity or stabilize the closed state, thereby reducing sensory-neuron hyperexcitability.

However, a wide variety of proteins, found in both neuronal and non-neuronal cells, are embedded in the plasma membrane, within the cell interior, and even in the extracellular environment, and contribute to the modulation of human pain. Importantly, there is only limited overlap between the pain-related targets currently under investigation and the new, potentially high-impact targets emerging from recent human genetic studies [5,6,7,8,9]. Large-scale biobank datasets that link genetic variation to pain-related phenotypes have made this gap increasingly evident, highlighting the need to broaden and update our target landscape.

Yet despite this wealth of emerging information, the practical constraints of time, funding, and the existing literature often push pain-target discovery toward the familiar. As a result, many researchers end up searching “under the lamppost,” focusing on well-trodden mechanisms while potentially overlooking genuinely promising new targets.

My hope is that the examples presented here will broaden, rather than narrow, the scope of current thinking. We urgently need more effective and innovative pain treatments, and expanding our view of viable drug targets is an essential step toward that goal.

## 4. Human Genetic Insight from Rare and Common Gene Variants

Pharmaceutical companies are increasingly drawing on large-scale human genetic resources such as the UK Biobank, the Million Veteran Program and FinnGen in hopes that directly linking a pain target to a pain phenotype will boost the probability of clinical success [5,6]. Recent estimates indicate that genetically supported targets can more than double the likelihood that a drug will ultimately reach patients [10]. Yet, for pain specifically, a similarly comprehensive analysis of genetics and novel pain therapeutics has not been published.

Target selection remains the single most critical decision in any drug discovery program. As such, all evidence that can increase the probability of success or reduce the risk of costly failure is highly valuable. Human genetic data is particularly powerful in this regard, offering an early window into potential efficacy as well as unwanted safety liabilities.

Genomic evidence is crucial for drug discovery because it directly links a target to disease biology. The strongest support comes from genes with clear gain- or loss-of-function effects that offer a direct therapeutic hypothesis. When only protective or high-impact variants exist, functional validation is essential to confirm translatability. GWAS-based insights can guide target directionality, though their functional impact often requires deeper investigation.

However, several high-profile disappointments, most notably NaV1.7, NGF, and TRPA1, demonstrate that even strong genetic support does not guarantee successful translation into effective pain treatments. Canonical sodium channel targets such as NaV1.7 (SCN9A) and NaV1.8 (SCN10A) derive their validation largely from rare variants: gain-of-function mutations that increase pain and loss-of-function mutations that lead to pain insensitivity. These extreme phenotypes provide compelling evidence for their central role in nociception.

Curiously, some NaV1.7 variants previously thought to be pain-causing are relatively common in UK Biobank genome-wide association studies but show no increased risk of chronic or neuropathic pain. Population-level prescribing patterns also reveal no elevation in the use of opioid or anti-neuropathic pain medications in carriers [11]. These findings suggest that a single NaV1.7 gain-of-function variant may not be sufficient to drive pain in the general population.

Overall, emerging genetic evidence points toward a more intricate architecture of chronic pain. Rather than being driven by single variants, pain risk likely arises from the combined influence of many common variants that confer broad polygenic susceptibility, alongside rare variants that exert large, sometimes decisive, effects. Increasingly, studies show convergence between these two classes of genetic contributors [12].

## 5. From Transcriptomes to Function: How Multichannel Sodium Signaling Shapes Sensory Neuron Excitability

Transcriptomics, the study of all RNA molecules in a cell or tissue, helps reveal gene expression patterns under different conditions. Recent transcriptomic analyses of human dorsal root ganglion (DRG) neurons and non-neuronal cells have produced a harmonized DRG atlas, which is now freely available online [13,14,15]. This enables detailed exploration of gene expression in specific sensory neuron subtypes and across all DRG cell types. However, data from healthy individuals alone provide a limited view, as only comparisons between healthy and diseased states can reveal genes that are differentially regulated in neuronal and non-neuronal pain pathways. It is likely that RNAseq data from diseased and well-phenotyped cohorts of human DRGs will be published in the near future.

However, challenges remain:

- mRNA levels do not always correlate with protein abundance or localization.
- Protein interactions within polarized sensory neurons are still poorly understood.

A major insight from recent work is the surprisingly broad expression of NaV channel subtypes, NaV1.1, NaV1.2, NaV1.3, NaV1.5, NaV1.6, NaV1.7, NaV1.8, and NaV1.9, within pain-sensing neurons. This widespread distribution indicates substantial functional redundancy: multiple channels can support similar electrophysiological roles, allowing one subtype to compensate when another is blocked. Although each NaV channel has its own biophysical characteristics, all are capable of generating action potentials when neurons are depolarized. Historically, NaV1.7 and NaV1.8 were thought to be the principal drivers of axonal conduction, but the broader expression landscape challenges this view.

This redundancy provides a compelling explanation for the limited analgesic efficacy of suzetrigine and suggests that targeting a single NaV subtype may be insufficient for robust pain relief. In contrast, non-selective NaV channel blockade, such as with lidocaine, can completely eliminate neuropathic pain, even when central sensitization is already established [16]. Reinforcing this concept, recent evidence shows that NaV1.5, a cardiac-specific sodium channel, is also expressed in sensory neurons and contributes to mechanical pain [17].

Because systemic inhibition of NaV1.5 is not clinically tolerable, selectively blocking only NaV1.7, NaV1.8, or NaV1.9 cannot fully reproduce the broad suppression of excitability achieved by multi-channel blockade. Together, these findings clarify why subtype-restricted interventions yield only modest analgesia and highlight the therapeutic potential of strategies that more comprehensively reduce peripheral NaV channel activity.

## 6. Multiomic Insight from Common Diseases: Osteoarthritis Pain as a Case Study

A growing body of epidemiological evidence indicates that obesity is a major driver of the global increase in osteoarthritis (OA) [18]. Excess body weight does more than overload the joint surface; it also increases mechanical stress on tendons and ligaments, structures increasingly recognized as contributors to inflammatory arthritis [19].

These insights raise a timely and important question: Could obesity-induced low-grade inflammation and metabolic dysregulation within tendons, ligaments, and the synovium, the metabolically active lubricating membrane lining joint capsules and tendon sheaths, initiate and sustain chronic OA pain?

This hypothesis is strengthened by the observation that synovial inflammation is highly prevalent across all stages of OA, from early disease to advanced degeneration [20]. Together, these findings point toward a model in which obesity not only increases mechanical joint loading but also creates a pro-inflammatory, metabolically altered microenvironment that may accelerate OA progression and amplify pain.

Multiomic analyses of human diseased tissues may offer a powerful route to identifying novel pain targets for highly prevalent diseases like OA. By integrating cell–type–specific transcriptomic, proteomic, and metabolomic data, it may be possible to pinpoint the biological processes that both initiate and maintain pain.

Recent human multiomic and genetic studies have begun to map these mechanisms. One large-scale analysis identified eight major biological processes implicated in OA pathogenesis, including the circadian clock, glial-related pathways, and TGFβ, FGF, WNT, BMP and retinoic acid signaling, as well as extracellular matrix organization [7]. Complementary work has shown that multiple immune cell types, such as dendritic cells, monocytes, macrophages, fibroblasts, and osteoclasts, are enriched in OA-affected synovium [21], underscoring the importance of inflammation in joint degeneration.

Clinical evidence further underscores the central role of peripheral tissues in driving OA pain. Patients with advanced, painful OA typically experience substantial and durable pain relief following joint replacement surgery, indicating that simply removing the diseased and inflamed joint tissues is often sufficient to resolve symptoms. This strongly supports a model in which peripheral sensitization within the joint microenvironment sustains chronic OA pain.

Subchondral bone marrow lesion size has been shown to correlate with both the severity of weight-bearing pain and changes in pain intensity over time, independent of non-weight-bearing pain, in knee OA [22]. This relationship suggests that an increased number of osteoclasts within the affected joint may contribute not only to the formation of subchondral bone lesions but also directly to pain generation. Together, these findings emphasize the need for deeper investigation into the role of osteoclasts in the genesis of OA pain.

An important next step is understanding how joint pathology communicates with the nervous system. For example, it would be informative to know whether joint replacement alters immune-cell infiltration in dorsal root ganglia (DRG). Supporting this line of inquiry, recent work showed that systemic macrophage depletion reduced pro-inflammatory macrophages in the DRG and alleviated pain behaviors in a surgically induced OA model without affecting joint damage [23]. These findings highlight immune–neuron interactions as potential drivers of chronic OA pain.

Intriguingly, metformin use in patients with type 2 diabetes has been associated with reduced risk of total joint replacement in OA patients, hinting at a protective effect [24]. Blocking NaV1.7 in chondrocytes has been shown to be chondroprotective [25], and earlier work demonstrated that metformin downregulates NaV1.7 expression via the ubiquitin ligase NEDD4-2 [26]. Together, these results suggest that metformin may partly help protect chondrocytes through reduced NaV1.7 channel expression.

Because disease modification alone is insufficient for regulatory approval and meaningful pain relief remains essential, the critical challenge is to identify the biological processes that specifically drive OA pain, rather than OA pathology in general. A recent human multi-omic study integrating transcriptomic and genetic data provides important clues: the peptidergic C fiber subtype hPEP.PIEZO and the Aδ low threshold mechanoreceptor subtype hAd.LTMR appears to contribute to joint pain and knee pain, respectively [27]. Notably, both neuronal subtypes are specialized for detecting mechanical pain, aligning well with the predominantly mechanical nature of OA symptoms.

Additional evidence comes from clinical studies showing that intra-articular administration of TRPV1 agonists provides significant pain relief in OA patients [28]. This observation is consistent with the expression of the TRPV1 ion channel in peptidergic C-fiber sensory neurons, further supporting the idea that targeting specific nociceptor subtypes may offer a path to effective analgesia in OA.

## 7. Human In Vitro Electrophysiology, Target Engagement and Clinical Analgesia

Access to human dorsal root ganglion (DRG) neurons from organ donors has significantly advanced pain research. These sensory neurons can now be obtained either from deceased organ donors or from patients undergoing specific surgical procedures [29].

The whole cell patch clamp technique enables measurement of ion channel currents and real-time membrane voltage in live cells [30]. In voltage clamp recordings from human DRG neurons, suzetrigine was shown to block human NaV1.8 channels with an IC_50_ of 0.68 nM [2]. In current clamp recordings, the selective NaV1.8 blocker suzetrigine reduced repetitive action potential firing in a dose-dependent manner during depolarization, but did not completely eliminate it. Remarkably, neurons were still capable of firing action potentials even when more than 99% of NaV1.8 channels were inhibited [31]. Dynamic clamp studies have shown that reducing NaV1.8 conductance substantially alters DRG neuron excitability, both under baseline conditions and in neurons rendered hyperexcitable by a NaV1.7 mutation associated with neuropathic pain. Notably, a subset of nociceptors displayed only a weak response to NaV1.8 subtraction, suggesting that additional ion channels must be targeted to achieve comprehensive pain relief [32].

Pharmacodynamic–pharmacokinetic (PK) correlations for suzetrigine can be established using PK studies conducted in animal models as well as in human volunteers and patients. These studies measure total and unbound suzetrigine concentrations in plasma, DRG tissue, and brain. An integrated FDA review document indicates that therapeutic doses of suzetrigine block approximately 90–95% of NaV1.8 channels, demonstrating a high degree of in vivo target engagement [1]. Target engagement refers to the extent to which a drug interacts with its intended molecular target in a living biological system.

These findings raise the possibility that achieving even closer to 99% NaV1.8 inhibition at clinically relevant exposures may reveal the full analgesic potential of NaV1.8 blockade. However, achieving such high levels of inhibition would likely require higher doses, potentially narrowing the therapeutic safety margin even if it results in stronger pain relief.

It is noteworthy that VX993, a follower compound to suzetrigine, appears to have been optimized for improved solubility while maintaining high potency and selectivity for NaV1.8. In general, increased solubility can be expected to achieve higher target engagement in vivo. Despite this, VX993 did not produce statistically significant relief of acute pain in clinical testing [3]. Improved solubility typically enhances absorption, distribution and excretion but reduces metabolism (ADME) properties relative to its predecessor. However, a potential drawback is that increased solubility may reduce penetration into the peripheral nervous system (PNS) and central nervous system (CNS).

P glycoprotein (P-gp) is an important factor in this context, as it limits CNS exposure for many drugs. At the blood–brain barrier, P-gp actively transports small-molecule xenobiotics out of endothelial cells and back into the bloodstream, creating an “efflux barrier” that restricts passive diffusion into the brain.

## 8. Species Differences in NaV1.8 Current Density and NaV Blockade Site of Action

An additional complication in drawing conclusions from in vivo studies performed in mouse and rat: when human NaV1.8 is expressed in mouse NaV1.8 knockout DRG neurons, its peak current is about twofold larger, action potential duration is about three times longer and shows increased firing frequencies compared with mouse DRG neurons [33]. This suggests that human NaV1.8 requires more substantial inhibition to achieve analgesia.

Finally, the site of action matters. Pharmacokinetic data show that suzetrigine readily penetrates the CNS, achieving comparable concentrations in the brain and plasma [1]. This distribution strongly suggests that suzetrigine can access and block NaV1.8 channels not only in peripheral axons but also in the central axons of these neurons, enabling balanced, pathway-wide NaV1.8 inhibition.

This distinction is critical. If NaV1.8 blockade is restricted to peripheral nerve endings, axons, or even the sensory neuron soma, NaV1.8-dependent action potentials can re-emerge once sufficiently strong depolarization reaches the central axons, allowing pain signals to continue propagating. In contrast, a compound like suzetrigine, capable of inhibiting NaV1.8 across the entire nociceptive pathway, has the potential to achieve a far more complete and durable suppression of pain signaling.

Interestingly, an elegant study using a human DRG–spinal cord ex vivo preparation showed that suzetrigine did not reduce capsaicin-evoked CGRP release from central terminals. In contrast, it was found that suzetrigine robustly suppresses CGRP release when applied to the DRG or nerve root. Together, these findings argue against a major role for central NaV1.8 in regulating pain-signal transmission within the spinal cord [34].

A selective NaV1.7 blocker, PF 05089771, developed by Pfizer, failed to demonstrate clinically meaningful analgesia in patients with painful diabetic neuropathy [35]. PF 05089771 carries a positive charge at physiological pH, a property known to limit CNS penetration and likely contributing to its lack of efficacy in humans.

**Figure 1:**
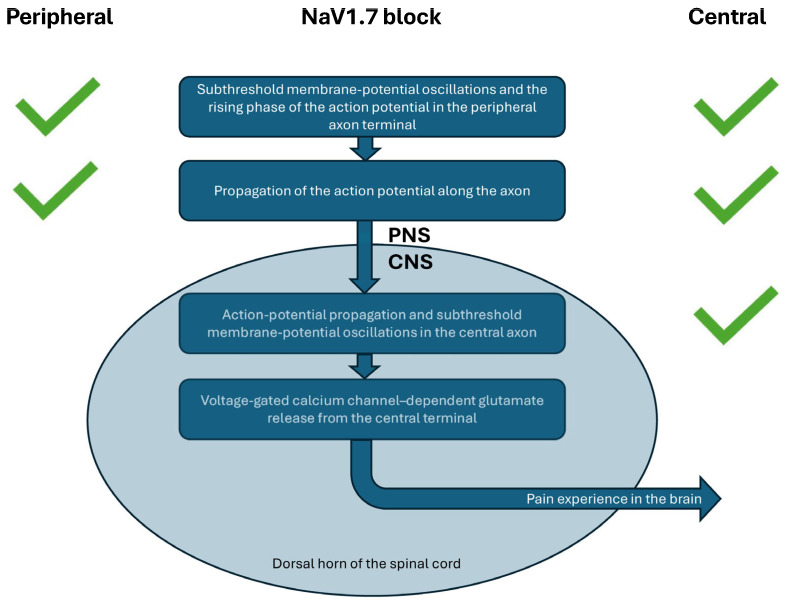
Differences Between Peripheral and Central NaV1.7 Block. A peripherally restricted NaV1.7 blocker reduces subthreshold membrane potential oscillations and action potential initiation in peripheral nerve terminals. It also limits action potential propagation along peripheral axons. A centrally acting NaV1.7 blocker, by contrast, produces a broader inhibition profile. It blocks NaV1.7-dependent action potential propagation and subthreshold oscillations within central axons as well. Importantly, peripheral NaV1.7 block has no effect beyond the PNS–CNS junction. The passive electrotonic spread that reaches into the CNS is too weak to depolarize central terminals enough to open voltage-gated calcium channels. Without sufficient calcium entry, the calcium-dependent mechanisms that drive glutamate release in central synaptic terminals cannot occur. Action potentials are required to generate the level of depolarization needed to activate these channels and initiate neurotransmitter release.

However, new evidence challenges the long-held assumption that peripheral NaV1.7 block alone is sufficient for pain relief. A recent study showed that intrathecal administration of PF 05089771 produced robust analgesia across multiple animal pain models [36]. This finding highlights the importance of drug access to central NaV1.7 channels (Figure 1).

Further supporting this concept, intrathecal delivery of a NaV1.7 antisense oligonucleotide resulted in significant pain relief along with a marked reduction in NaV1.7 protein in the dorsal horn, DRG soma, and central axons but not in peripheral nerve fibers [37]. These data strongly support the hypothesis that central inhibition of NaV1.7 is both necessary and sufficient to produce analgesia, whereas peripherally restricted compounds may be inherently limited.

Another limitation of selective, peripherally restricted NaV1.7 channel blockade is that NaV1.7 is also expressed in autonomic nerves that regulate blood pressure. As a result, selective inhibition of NaV1.7, such as with MK-2075, has been shown to cause orthostatic hypotension and even syncope in healthy volunteers [38].

Clinical and preclinical data from suzetrigine and PF 05089771 help explain why VX993 failed in the acute pain clinical trial. VX993 was likely designed to improve solubility, allowing its use as an intravenous formulation. However, greater solubility is likely to reduce penetration into the CNS and PNS. Although small-molecule compounds that enter the CNS typically also reach the DRG, the contribution of P-gp–mediated efflux in DRG has traditionally been considered less significant than in the CNS [39]. However, new single-cell RNA-seq data reveal that a subset of sensory neurons, endothelial cells, and injury-associated ATF3-expressing neurons also express ABCB1, the gene encoding P-gp [14]. This suggests that P-gp may create local micro-gradients for potential substrates such as VX-993 and could reduce its effective concentration within specific DRG compartments.

I propose the hypothesis that limited CNS/PNS exposure by VX993 contributed to its lack of analgesic efficacy.

## 9. Why the Peripheral TRPA1 Blocker Did Not Provide Pain Relief?

TRPA1 is a nonselective cation channel expressed in pain-sensing neurons [40]. It is activated by a wide range of known pain mediators [41], and activation in human volunteers reliably produces pain [42]. Moreover, gain-of-function variants in the TRPA1 gene are genetically associated with increased pain sensitivity in humans [8,43].

Despite extensive drug discovery efforts to develop potent, selective, and drug-like TRPA1 antagonists, clinical translation has been challenging. Eli Lilly evaluated a peripherally restricted TRPA1 antagonist, LY3526318, in patients with diabetic neuropathic pain, osteoarthritis pain, and low back pain [44]. Unfortunately, LY3526318 did not provide meaningful pain relief [45].

However, a growing body of preclinical evidence suggests that peripheral blockade alone may not be sufficient. TRPA1 is also expressed in the central axons of peripheral sensory neurons, where its activation amplifies nociceptive signaling [46,47]. Importantly, intrathecal administration of a small dose of a selective TRPA1 antagonist, one incapable of blocking peripheral TRPA1, produced robust analgesia in animal models [48,49].

Initial Northern blot analysis of bulk RNA suggested that TRPA1 expression was restricted to the peripheral nervous system [40]. In contrast, recent single-cell RNA seq analyses reveal TRPA1 expression in the human brain and spinal cord [50]. In animal studies, TRPA1 activation within the amygdala in the brain has been shown to enhance both nociceptive responses and the affective dimension of pain [51].

Taken together, these findings strongly suggest that the full analgesic potential of TRPA1 antagonism remains untested in clinical trials. A compound capable of blocking TRPA1 in both peripheral and central axons of pain-sensing neurons and in the CNS may be required to unlock the true therapeutic benefit of this target.

## 10. Animal Models

Animal models have historically played a critical role in the development of novel painkillers [52]. Only an awake animal can exhibit pain-like behaviors, enabling in vivo assessment of potential analgesic efficacy. However, reproducibility of the in vivo results is still a major issue. This has led to requests from scientific journals for more transparent reporting of data, use of blinding and randomization, and other good practices to avoid bias. An interesting new development is the use of machine vision and machine learning to automatically extract and quantify behavioral features that capture the internal pain state of rodents in multiple pain models [53], and a machine learning tool with light-based image analysis for automatic classification of 3D pain behaviors [54], which hopefully could provide unbiased behavioral data with minimal human interference.

Today, one of the main reasons for conducting in vivo animal studies is to gain insight into target engagement and establish pharmacokinetic/pharmacodynamic (PK/PD) correlations. In simple terms: How much of the target protein needs to be blocked in vivo based on the in vitro IC_50_ value?

The development of suzetrigine illustrates several key issues. Suzetrigine is a highly potent and selective NaV1.8 blocker. According to FDA documentation, suzetrigine was advanced to clinical trials without prior animal efficacy testing, though pharmacokinetics and toxicology were studied in animals. Why [1]?

A recent study showed robust efficacy of suzetrigine in a mouse neuropathic pain model, despite the fact that suzetrigine blocks mouse NaV1.8 nearly 500 times less potently than human NaV1.8 [55]. This implies that achieving efficacy in mice would require doses hundreds of times lower than those used in humans. Clearly, a mouse PK–PD study is not ideal for human dose prediction.

Vertex made a bold decision to proceed directly to human trials without animal efficacy support, relying instead on pharmacokinetic modeling to predict human exposure and safety margins. FDA data now confirm that NaV1.8 is inhibited by ~95% at therapeutic doses in humans. This sheds light on the mouse data:

- In mice, analgesia occurred at exposures well below the IC_50_, whereas in humans, nearly complete NaV1.8 blockade was required.
- This highlights the danger of drawing conclusions from animal efficacy studies without accounting for free, unbound drug concentrations and species-specific pharmacology.

One key reason animal models remain indispensable is their ability to capture the full, integrated physiological response to drug exposure across intended molecular targets, biochemical pathways, neuronal circuits, and anatomical sites that collectively shape pain processing in vivo. This systems-level response simply cannot be inferred from in vitro studies, which isolate individual components and therefore miss emergent network effects.

Moreover, some drugs are converted into pharmacologically active metabolites in the body. These metabolites can significantly amplify or modify the net analgesic effect, and their contribution can only be accurately assessed through in vivo experimentation.

## 11. Conclusions

Human genetic insights from large-scale biobanks continue to offer a powerful and unbiased route for discovering new pain targets. As these datasets expand, it is increasingly likely that the next wave of genetically supported pain targets will emerge not only from direct associations with pain phenotypes but also from genes embedded in biologically relevant pathways connected to pain phenotypes through genetic linkage. The primary bottleneck is no longer in generating genetic insights, but in transforming those insights into actionable and therapeutically meaningful targets.

Crucially, incorporating genetic data from individuals with more diverse ancestries will substantially increase the statistical power to detect previously overlooked pain-driving genes [6]. This broader genetic representation strengthens confidence in target relevance and enhances the generalizability of findings.

At the same time, advances in multi-omics, particularly RNA seq, allow researchers to map gene expression across the extended pain pathway, identifying targets enriched not only in sensory neurons but also in key non-neuronal cell types that modulate nociception. The goal remains clear: to prioritize targets selectively enriched in these pain-relevant circuits while minimizing the risk of unwanted effects in unrelated tissues.

About 98–99% of human genetic variation occurs in non-coding regions of the genome, which include key regulatory elements such as enhancers, promoters, and transcription factor–binding sites [56,57]. Many quantitative trait loci (QTLs) are genomic regions whose DNA variants influence continuously varying traits. QTLs act by regulating gene and protein expression rather than altering protein structure. Because of this, high-resolution quantitative proteomic analyses of healthy and diseased human tissues provide a powerful opportunity to identify the proteins and molecular pathways that drive and maintain chronic pain.

The rapid emergence of new therapeutic modalities, ranging from biologics, antibody drug conjugates, PROTACs and molecular glues to RNA targeting agents, opens unprecedented opportunities to modulate target protein function, expression, or mRNA within the extended pain pathway that were previously inaccessible with small molecules alone.

## 12. Future Directions

**Figure 2:**
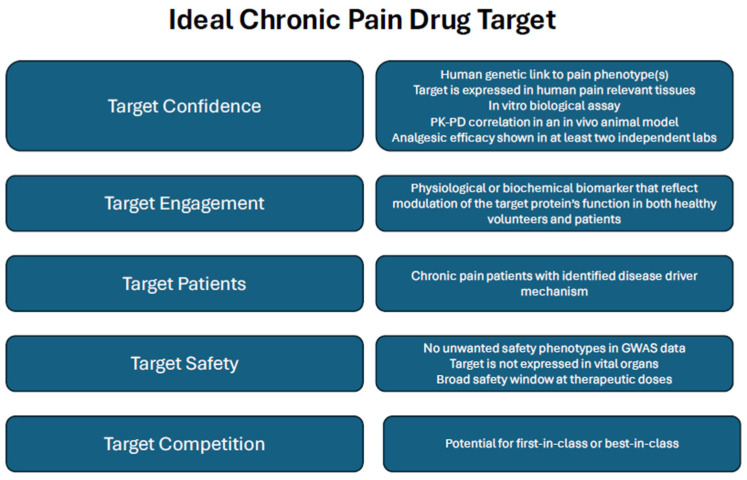
A five-dimensional framework for an ideal chronic pain drug target, adapted from Morgan et al. [58]. At the outset of a drug discovery project, several knowledge gaps typically remain. By the time the project is ready to nominate a candidate compound, most dimensions of the framework have been investigated and are supported by experimental data.

Ultimately, even as advanced genomic, transcriptomic, and proteomic tools continue to refine our understanding of pain biology, true progress depends on assembling a rigorous and persuasive pain target validation package (Figure 2) [58]. Such a framework must clearly define where the therapeutic is expected to act, establish why that anatomical or molecular site is essential to pain processing, and demonstrate how modulating the target is likely to produce clinically meaningful analgesia in humans. Equally crucial is determining whether strong human genetic evidence supports the candidate protein or the regulatory pathways that govern its activity or expression as a causal contributor to pain. Together, these elements provide the foundation needed to bridge fundamental discovery with translational success and to advance the next generation of pain therapeutics.

### 12.1. Limitations of Genetic Validation

Loss-of-function (LoF) variants are far more common in humans than previously recognized. The average person carries dozens of these gene-disrupting changes, including some that fully inactivate both copies of a gene. Although most LoF variants remain rare, a surprising number of genes can be completely knocked out in large segments of the population without causing any apparent health issues [59]. This widespread tolerance highlights the remarkable redundancy and built-in resilience of human biology.

However, genetic tolerance does not always translate to pharmacological tolerance. Developmental compensation can mask certain gene-loss effects in humans, meaning that naturally occurring LoF carriers may not fully mimic the biological consequences of drug-induced inhibition. For example, individuals with NaV1.7 loss-of-function do not exhibit the autonomic dysfunction that emerges when the same channel is selectively blocked by drugs, demonstrating that gene knockout and acute pharmacological inhibition can produce fundamentally different physiological outcomes [38].

Even genetically validated pain targets can fail in clinical trials, and several factors may contribute to these disappointments. For example, some genetically associated chronic pain targets, such as the Netrin-1 receptor DCC, may alter the wiring of pain-processing axonal circuits during brain development, thereby increasing susceptibility to multi-site chronic pain [9]. Developmental mechanisms like these are extremely challenging, if not impossible, to reverse with pharmacological interventions later in life.

In addition, disease-associated human gene variants may be mislocalized within cells or may activate signaling pathways that differ from those of the wild-type protein. When available, highly selective antibodies can be invaluable: they enable visualization of the tissue distribution of both wild-type and mutant proteins. Analysis of downstream signaling can strengthen or weaken confidence that a given variant is genuinely disease-causing.

When protein-level tools are lacking, validating published RNA-seq findings with RNAscope, especially from patient tissues, can help increase confidence in target expression patterns, even though mRNA and protein levels rarely show a one-to-one relationship. In parallel, genetic deletion of the target, for example, using siRNA to mimic a LoF state, can further support target validation by providing functional evidence that aligns with human genetics.

Algorithms that predict the functional consequences of amino acid substitutions in missense variants or even the effects of protein-truncating mutations should ideally be supported by experimental validation. Computational predictions provide useful first insights, but they cannot yet fully capture the complexity of protein function in a biological context. A striking example comes from studies of a truncated human TRPA1 variant, which unexpectedly exhibits gain-of-function activity when co-expressed with the wild-type channel, rather than the anticipated LoF [60].

GWAS hits often highlight genomic regions without immediately clarifying which specific gene is functionally involved. Although modern analytical methods improve gene prioritization at each locus, their accuracy is still evolving. Importantly, current evidence increasingly shows that the gene closest to the association signal is often the most relevant.

### 12.2. Limitations of Translational Validation

Species differences present another major challenge. Human target proteins often differ significantly in amino acid sequence from those of commonly used laboratory species, limiting the predictive value of many preclinical models. In contrast, non-human primates generally show much higher sequence homology and therefore more reliably reproduce human primary pharmacology. However, the use of non-human primates in experimental pain studies is prohibited in the EU, and their use requires strict scientific and ethical justification worldwide.

In vitro assays usually evaluate recombinant proteins from multiple species to assess cross-species differences in primary pharmacology. However, small-molecule metabolism and metabolite profiles can also vary substantially across species, meaning that the choice of experimental animal often ultimately depends on whether adequate drug exposure and metabolite profile can be achieved in the target tissue.

In addition, some targets undergo alternative splicing. Tissue-specific splice variants may differ across species in both structure and expression levels, potentially resulting in divergent pharmacological responses. Modern long-read mRNA sequencing technologies now enable far more accurate characterization of these splice variants in both human and preclinical animal tissues, improving the selection and interpretation of relevant models

Together, these considerations underscore the importance of carefully selecting the most appropriate species for experimental target-validation studies.

### 12.3. Limitations of Clinical Validation

Historically, some clinical failures occurred simply because drug exposure in the relevant target tissues was insufficient. Today, this risk is far lower thanks to comprehensive pharmacokinetic assessments and robust human exposure predictions. A widely used risk-mitigation strategy is to design clinical dosing regimens that achieve at least IC_90_ target engagement.

Finally, clinical programs may fail when the chosen pain indication does not align with the biological function of the target. Only a limited number of pain conditions have clinically validated endpoints and study protocols, making indication selection particularly challenging. A promising way to manage this uncertainty is to evaluate several high-potential indications in parallel. Although this approach increases development costs, it significantly reduces the risk of investing in the wrong indication.

## Data Availability

No new data were created or analyzed in this study. Data sharing is not applicable to this article.

## Abbreviations

ABCB1	A gene encoding P-glycoprotein
ADME	Absorption, Distribution, Metabolism, Excretion
ASIC	Acid-Sensing Ion Channel
ATF3	Activating Transcription Factor 3
BMP	Bone Morphogenetic Protein
CNS	Central Nervous System
CGRP	Calcitonin-Gene Related Peptide
DCC	Deleted in Colorectal Carcinoma
DRG	Dorsal Root Ganglion
EU	the European Union
FDA	U.S. Food and Drug Administration
FinnGen	Large-scale Finnish public–private genomics research project
FGF	Fibroblast Growth Factor
GWAS	Genome-Wide Association Study
hAD.LTMR	Human A-delta low threshold mechanoreceptor subtype
HCN	Hyperpolarization-Activated Cation Channel
hPEP.PIEZO	Human peptidergic Piezo2-enriched sensory neuron subtype
IC_50_	Half maximal inhibitory concentration
IC_90_	Concentration that produces 90% inhibition
LoF	Loss-of-function
NaV	Voltage-gated sodium channel
NEDD4-2	E3 ubiquitin-protein ligase belonging to the NEDD4 family
NGF	Nerve Growth Factor
NNT	Number Needed to Treat
OA	Osteoarthritis
PD	Pharmacodynamics
P-gp	P-glycoprotein
Piezo2	A mechanosensitive non-selective cation channel
PK	Pharmacokinetics
PNS	Peripheral Nervous System
PROTAC	Proteolysis-Targeting Chimera
QTL	Quantitative Trait Loci
RNA	Ribonucleic Acid
siRNA	Small Interfering RNA, Double-Stranded Non-Coding RNA
SNRI	Serotonin-Norepinephrine Reuptake Inhibitor
TGFβ	Transforming Growth Factor-β
TRP	Transient Receptor Potential
TRPA1	Transient Receptor Potential A1
WNT	WNT Signaling Pathway. WNT is an amalgam of wingless and int.
